# Health-related quality of life in the randomized phase 3 study of ramucirumab plus docetaxel versus placebo plus docetaxel in platinum-refractory advanced urothelial carcinoma (RANGE)

**DOI:** 10.1186/s12894-020-00752-w

**Published:** 2020-11-07

**Authors:** Andrea Necchi, Hiroyuki Nishiyama, Nobuaki Matsubara, Jae-Lyun Lee, Daniel P. Petrylak, Ronald de Wit, Alexandra Drakaki, Astra M. Liepa, Huzhang Mao, Katherine Bell-McGuinn, Thomas Powles

**Affiliations:** 1 Department of Medical Oncology, Fondazione IRCCS Istituto Nazionale Dei Tumori, 20133 Milan, Italy; 2 University of Tsukuba, Tsukuba, Japan; 3 National Cancer Center Hospital East, Chiba, Japan; 4 Asan Medical Center, University of Ulsan College of Medicine, Seoul, Republic of Korea; 5 Yale University, New Haven, CT USA; 6 Erasmus MC Cancer Institute, Rotterdam, Netherlands; 7 University of California Los Angeles, Los Angeles, CA USA; 8 Eli Lilly and Company, Indianapolis, IN USA; 9 Barts Cancer Institute, London, UK

**Keywords:** Antiangiogenesis, Bladder cancer, Neoplasm metastatsis, Patient-reported outcomes, Quality of life, Ramucirumab, Urinary bladder neoplasm, Urothelial carcinoma

## Abstract

**Background:**

To evaluate patient-reported outcomes with ramucirumab plus docetaxel, a regimen which improved progression-free survival in platinum-refractory advanced urothelial carcinoma (aUC).

**Methods:**

RANGE—a randomized, double-blinded, phase 3 trial in patients with platinum-refractory aUC. Ramucirumab (10 mg/kg) plus docetaxel (75 mg/m^2^) or placebo plus docetaxel were administered every 21 days until disease progression or unacceptable toxicity. Patients received maximum 10 cycles of docetaxel. European Organization for Research and Treatment of Cancer Quality of Life Questionnaire C30 (EORTC QLQ-C30) and EuroQoL five-dimensions (EQ-5D-5L) were administered at baseline, start of each cycle, and 30-day follow-up visit. A ≥ 10-point change in QLQ-C30 scores was considered meaningful. Rates of improved/stable scores were compared between treatment arms using Fisher’s exact test. Time to deterioration (TtD) was estimated and compared using Kaplan–Meier estimation and log-rank test.

**Results:**

Of the 530 patients, ~ 97% patients in each arm provided baseline QLQ-C30 data. On-treatment compliance was ≥ 88% for first 8 cycles. Mean baseline QLQ-C30 scores were similar between arms, with global quality of life (QoL), fatigue, pain, and insomnia having greatest impairment. Postbaseline rates of improved/stable QLQ-C30 scores were similar between treatment arms except for greater improvement in pain score with ramucirumab. TtD of QLQ-C30 scales favored ramucirumab arm. Baseline EQ-5D-5L index and visual analogue scale scores were similar between arms, followed by relatively stable on-treatment scores. EQ-5D-5L scores worsened at post-discontinuation follow-up visit.

**Conclusions:**

Ramucirumab plus docetaxel did not negatively impact QoL compared with docetaxel alone in platinum-refractory aUC. Improved TtD and tumor associated rates of pain favored ramucirumab treatment.

***Clinical trail registration*:**

NCT02426125. https://clinicaltrials.gov/ct2/show/NCT02426125. Date of registration: April 24th 2015

## Background

Advanced/metastatic urothelial carcinoma (UC), including carcinomas arising in the bladder, urethra, ureter, and/or renal pelvis, has a poor prognosis. Median survival is approximately 12–14 months with standard first-line platinum-based chemotherapy for advanced/metastatic disease [1]. Despite approvals of various targeted therapies for platinum-refractory advanced UC, most patients have limited treatment options as they experience disease progression. Ramucirumab plus docetaxel has shown higher response rate and improved progression-free survival (PFS) compared to placebo plus docetaxel in the platinum-refractory population [2, 3]. In the refractory setting, quality of life (QoL) is an important factor in the treatment decision-making process for patients with cancer where symptom palliation is the primary goal of therapy [4, 5]. Monitoring of QoL via patient-reported symptoms is also associated with better outcomes than observed with usual care [6].

UCs overexpress the vascular endothelial growth factor (VEGF) ligand and its receptor, vascular endothelial growth factor receptor (VEGFR)-2 [7, 8]. Ramucirumab (a human IgG1 monoclonal antibody and a VEGFR-2 antagonist) [9, 10] plus docetaxel improved PFS (hazard ratio [HR] = 0.757; 95% confidence interval [CI] [0.607, 0.943]) compared with placebo plus docetaxel in the randomized phase 3 trial for platinum-refractory advanced UC (RANGE; NCT02426125) [2]. Whereas other antiangiogenic drugs have failed thus far in refractory UC [1, 11, 12]. Patients treated in the ramucirumab arm also had a numerically higher objective response rate (24.50%, 95% CI [18.80, 30.30] vs 14.0%, 95% CI [9.40, 18.60]) [2]. Although not statistically significant, overall survival (OS) at the final analysis favored the ramucirumab arm (HR = 0.887; 95% CI [0.724, 1.086]; *p* = 0.25) [3]. The most frequent adverse events (AEs) of any grade reported for both the ramucirumab and placebo arms were fatigue (39.1% vs 36.2%), alopecia (23.6% vs 30.6%), diarrhea (23.6% vs 16.6%), decreased appetite (22.1% vs 17.0%), nausea (22.1% vs 14.0%), and stomatitis (23.2% vs 9.0%). Of these AEs, only fatigue had an incidence of ≥ 5% for grade 3/4 (7.0% vs 6.0%). On-study, treatment-related deaths were relatively rare (8 [3.0%] and 5 [2.0%], respectively) [3]. Overall, the proportion of patients with AEs receiving ramucirumab plus docetaxel was similar to the proportion of patients with AEs in the placebo plus docetaxel arm. No new safety signals were detected for ramucirumab plus docetaxel, and the safety profile was considered manageable [2].

The impact of treatment on QoL is not widely reported in refractory advanced UC (aUC), particularly for chemotherapy and anti-VEGFR-2 drug combination therapies. Therefore, we assessed the patient-reported outcomes (PROs) as secondary endpoints in the RANGE trial to evaluate the benefit-risk profile of the addition of ramucirumab to docetaxel from the patient perspective [3]. We hypothesized that patients would experience more symptomatic improvement without experiencing detriment in QoL.

## Methods

### Study design

The RANGE trial design and the outcome of its primary endpoint were previously published [2]. The trial was conducted during July 2015 and April 2017 and approved by appropriate review boards/ethics committees and followed the principles outlined in the Declaration of Helsinki. All patients provided written informed consent. This study adhered to the CONSORT guidelines.

### Patient eligibility criteria

Eligibility criteria included patients with Eastern Cooperative Oncology Group (ECOG) performance status of zero or one and whose advanced disease progressed during or ≤ 14 months after first-line platinum-based chemotherapy. Patients may have also received one checkpoint inhibitor therapy prior to enrollment onto RANGE, in which case prior platinum-containing chemotherapy in ≤ 24 months was allowed. Patients were excluded if they had received a taxane previously [2].

Eligible patients were randomized to receive docetaxel (75 mg/m^2^, intravenously [IV]) plus ramucirumab (10 mg/kg, IV) or docetaxel plus placebo, administered on day 1 of each 21-day cycle. Randomization was stratified by geographic region (North America, East Asia, or Europe/other), ECOG performance status at baseline (0 or 1), and visceral metastasis (present or absent). Ramucirumab treatment continued until there was documented disease progression, toxicity or intolerance requiring discontinuation, withdrawal of consent, or noncompliance. Docetaxel could continue, if no prespecified discontinuation criteria were met, for up to six cycles with an additional four cycles allowed if an adequate treatment response was observed. Tumor responses were assessed using Response Evaluation Criteria in Solid Tumors (RECIST) v1.1 [13].

### Outcomes and assessments

Assessment of PROs, a secondary endpoint of the RANGE trial, was conducted through use of the European Organization for Research and Treatment of Cancer Quality of Life Questionnaire C30 (EORTC QLQ-C30), version 3 and the EuroQoL five-dimensions, 5 level (EQ-5D-5L) questionnaire, which measure QoL (functional domains and symptoms) and health status, respectively [14, 15]. Patients completed the questionnaires only if there were cross-culturally validated translations in which they were fluent. Prior to any extensive contact with clinical personnel, patients were asked to complete the questionnaires at baseline, prior to the start of each cycle, and within 30 days after treatment discontinuation (follow-up). QLQ-C30 data were scored according to the EORTC guidelines, using a 0–100 scale with higher scores for global QoL and functional scales representing better QoL and lower scores for symptom scales representing less burden. A ≥ 10-point change was considered clinically meaningful [16]. EQ-5D-5L index values were based on the value set for England with a range of -0.281 to 1, with zero representing death and one representing perfect health [17]. Additionally, patients indicated their current health status by marking on a visual analogue scale (VAS) ranging from 0 (worst-imaginable health state) to 100 (best-imaginable health state) [15].

### Statistical analysis

Analyses were conducted in the intention-to-treat (ITT) population except for descriptive statistics that were limited to the number of patients who provided data at a given assessment. Compliance rates were calculated based on the number of patients expected to provide data at a given assessment (i.e., those patients still receiving study therapy). Descriptive statistics were used to summarize data, including change from baseline as well as for the EQ-5D-5L scores. QLQ-C30 scores were classified as improved or worsened if change from baseline was ≥ 10 points; change < 10 points was classified as stable. For each scale at each postbaseline assessment, the proportion of patients with improved or stable scores was compared using the Fisher’s exact test. Time to sustained deterioration (TtD) was defined as time from randomization date to first worsening of ≥ 10 points with no subsequent non-worsened assessment relative to baseline. If there were no subsequent assessments, the patient was classified as deteriorated. The follow-up assessment after treatment discontinuation could not be considered as a subsequent non-worsened assessment. Patients without deterioration were censored at the last non-deteriorated assessment. Kaplan–Meier method was used to estimate the probability of TtD, and unstratified log-rank test was used to investigate significance between treatment groups. Univariable Cox regression analysis was performed to test the association of treatment with TtD. No adjustments were made for multiplicity, but *p*-values < 0.05 were used to identify potential trends. These analyses discussed here were performed on the ITT population at the time of the OS database lock [3]. All the analyses were conducted using SAS software (SAS, Version 9.1.2 or higher).

## Results

### RANGE patients and questionnaire compliance

**Fig. 1:**
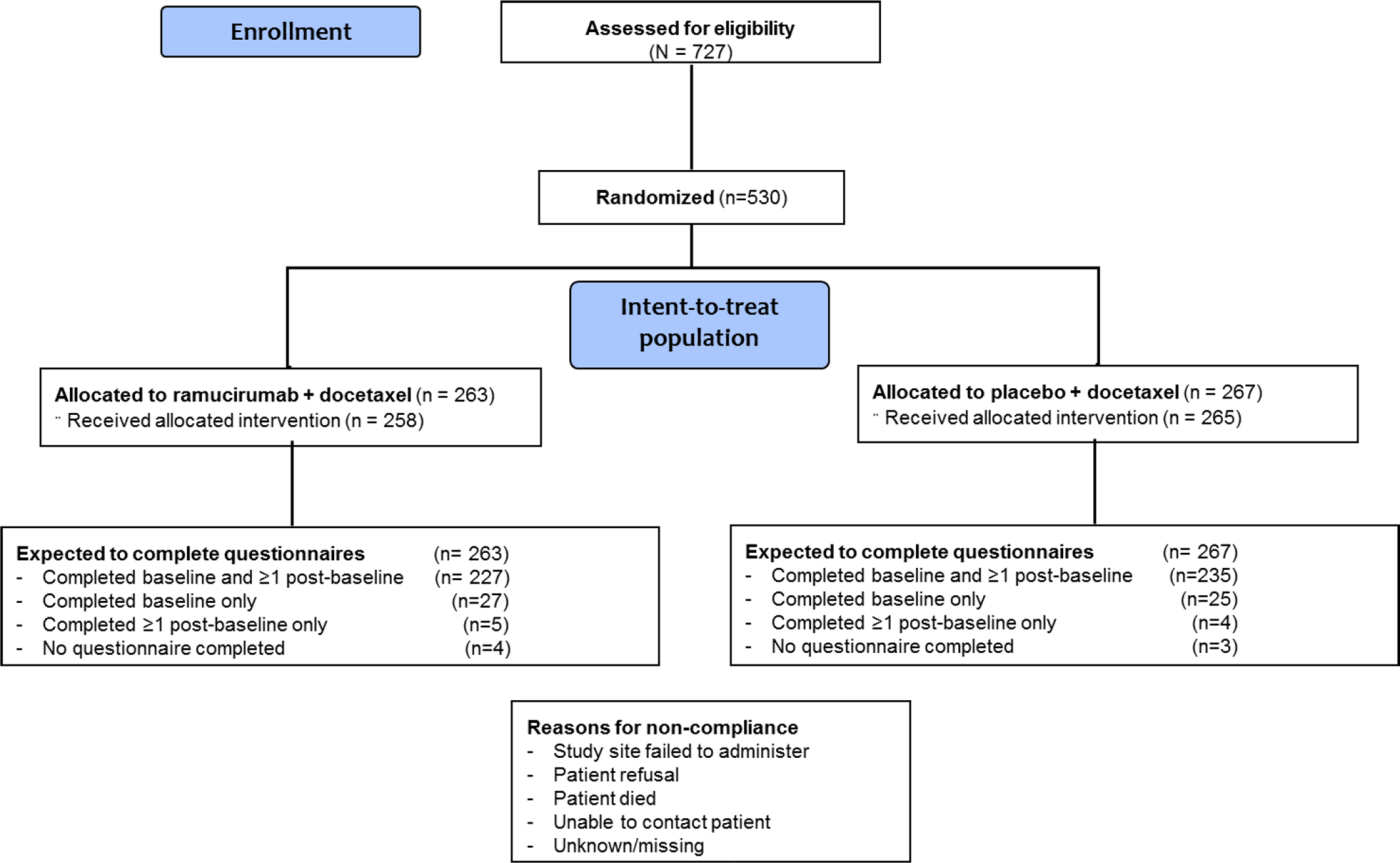
Patient disposition. *n* = number of patients

In the randomly assigned patients (N = 530, ITT population) from the 727 screened for eligibility (Fig. 1), baseline characteristics were similar between treatment arms (Table 1). At baseline, 254 (96.6%) of patients in the ramucirumab arm and 260 (97.4%) in the placebo arm provided QLQ-C30 and EQ-5D-5L data (Table 2). For the first 8 cycles, compliance was ≥ 88% for both the QLQ-C30 and EQ-5D-5L, (Table 2). The most common reasons for noncompliance were failure by the site to administer and patient refusal to complete the questionnaires (Fig. 1). At the post-discontinuation follow-up visit, compliance was lower overall, with 52–54% of patients providing data. When grouped by those who provided data at follow-up and those who did not, the groups were similar in baseline patient characteristics, time on therapy, BOR, and reasons for discontinuation.

**Table 1:** Baseline demographics and disease characteristics

	RAM + DOC (n = 263)	PL + DOC (n = 267)
*Median age, years (range)*	65 (34–86)	66 (32–83)
*Male*	213 (81)	215 (81)
*Race*		
White	203 (77)	204 (76)
Asian	54 (20)	61 (23)
*ECOG performance status*		
0	121 (46)	125 (47)
1	139 (53)	142 (53)
*Geography*		
North America	24 (9)	24 (9)
East Asia	53 (20)	57 (21)
Europe/Other	186 (71)	186 (70)
*Primary tumor site*		
Bladder	180 (68)	177 (66)
Urethra	7 (3)	6 (2)
Renal pelvis	39 (15)	42 (16)
Ureter	33 (13)	37 (14)
Other	1 (< 1)	5 (2)
*Sites of metastases*		
Lymph node only	41 (16)	42 (16)
Visceral	182 (69)	188 (70)
Liver	78 (30)	69 (26)
Lung	98 (37)	121(45)
Bone	56 (21)	53 (20)
*Hemoglobin < 10 g/dL*	34 (13)	36 (13)
*Patients with time since previous chemotherapy < 3mo*	115 (44)	126 (47)
*Bellmunt risk factors*		
0	88 (33)	93 (35)
1	105 (40)	109 (41)
2	64 (24)	57 (21)
3	6 (2)	8 (3)
*Prior neoadjuvant or adjuvant therapy*	87 (33)	107 (40)
*Prior platinum-based therapy:*		
Cisplatin	161 (61)	189 (71)
Carboplatin	97 (37)	77 (29)
*Prior immune checkpoint inhibitor*	17 (7)	28 (10)

*DOC* docetaxel, *ECOG* Eastern Cooperative Oncology Group, *mo* months, *n* number of patients, *PL* placebo, *RAM* ramucirumab

Data are n (%), unless otherwise indicated

For a full list of patient demographics and disease characteristics at baseline in the intention-to-treat population [2]

**Table 2:** EORTC QLQ-C30 and EQ-5D-5L compliance rates from baseline through cycle 8 and 30-day follow-up (ITT population)

Assessment time point	QLQ-C30		EQ-5D-5L	
	RAM + DOC (n = 263)	PL + DOC (n = 267)	RAM + DOC (n = 263)	PL + DOC (n = 267)
Baseline	254/263 (97)	260/267 (97)	254/263 (97)	260/267 (97)
Cycle 1	214/229 (93)	226/237 (95)	212/229 (93)	225/237 (95)
Cycle 2	159/165 (96)	159/165 (96)	158/165 (96)	159/165 (96)
Cycle 3	139/147 (95)	133/138 (96)	136/147 (93)	133/138 (96)
Cycle 4	114/126 (90)	100/105 (95)	114/126 (90)	100/105 (95)
Cycle 5	108/114 (95)	88/94 (94)	107/114 (94)	88/94 (94)
Cycle 6	79/87 (91)	64/71 (90)	83/87 (95)	63/71 (89)
Cycle 7	64/68 (94)	52/59 (88)	66/68 (97)	52/59 (88)
Cycle 8	52/56 (93)	36/41 (88)	51/56 (91)	36/41 (88)
Follow-up	116/215 (54)	119/231 (52)	113/215 (53)	121/231 (52)

*DOC* docetaxel, *EORTC QLQ-C30* European Organization for Research and Treatment of Cancer Quality of Life Questionnaire C30, *EQ-5D-5L* EuroQoL five-dimensions, *ITT* intention-to-treat, *PL* placebo, *RAM* ramucirumab

Data are presented as number/total number (%)

For compliance, the total number is the number expected to complete at the assessment time point

On-treatment compliance reporting is truncated at cycle 8, but similar rates were reported for later cycles

The median number of cycles administered in both treatment arms was 4

### Descriptive summaries

Mean baseline scores for both questionnaires were similar between treatment arms (Table 3). With high scores being favorable for global QoL and functional domains of the QLQ-C30, the lowest mean scores were reported for global QoL, indicating the domain with greatest impairment at baseline. With low scores being favorable for symptoms, the highest mean scores were reported for fatigue, pain, and insomnia, indicating the symptoms of greatest burden at baseline.

**Table 3:** Baseline Scores (Mean [standard deviation])

	RAM + DOC (n = 263)	PL + DOC (n = 267)
EORTC QLQ-C30 Scales^a^		
Global QoL	62.0 (22.9)	60.8 (21.4)
Physical functioning	75.9 (22.4)	74.2 (21.7)
Role functioning	73.3 (29.5)	72.1 (29.9)
Emotional functioning	76.0 (22.4)	75.0 (21.2)
Cognitive functioning	85.9 (18.3)	84.6 (20.3)
Social functioning	74.6 (28.8)	71.2 (30.2)
Fatigue	33.1 (25.9)	36.0 (24.8)
Nausea/vomiting	8.0 (16.1)	9.0 (17.5)
Pain	32.0 (31.1)	33.8 (30.7)
Dyspnea	17.4 (24.6)	17.9 (25.4)
Insomnia	27.7 (30.9)	28.8 (29.9)
Appetite loss	22.4 (29.2)	23.3 (31.9)
Constipation	21.0 (28.3)	24.6 (30.0)
Diarrhea	8.1 (16.6)	6.3 (16.0)
EQ-5D-5L Index Score^b^	0.77 (0.23)	0.78 (0.19)
EQ-5D-5L VAS	67.2 (21.7)	67.3 (18.7)

*DOC* docetaxel, *EORTC QLQ-C30* European Organization for Research and Treatment of Cancer Quality of Life Questionnaire C30, *EQ-5D-5L* EuroQoL five-dimensions, *n* number of patients, *PL* placebo, *QoL* quality of life, *RAM* ramucirumab, *SD* standard deviation, *VAS* visual analogue score

^a^For the EORTC QLQ-C30, high scores are favorable for functional domains and global QoL; low scores are favorable for symptoms

^b^For the EQ-5D-5L, high scores are favorable. The range for Index Score is -0.281 to 1 and the range for VAS is 0 to 100 (higher scores are favorable)

**Fig. 2:**
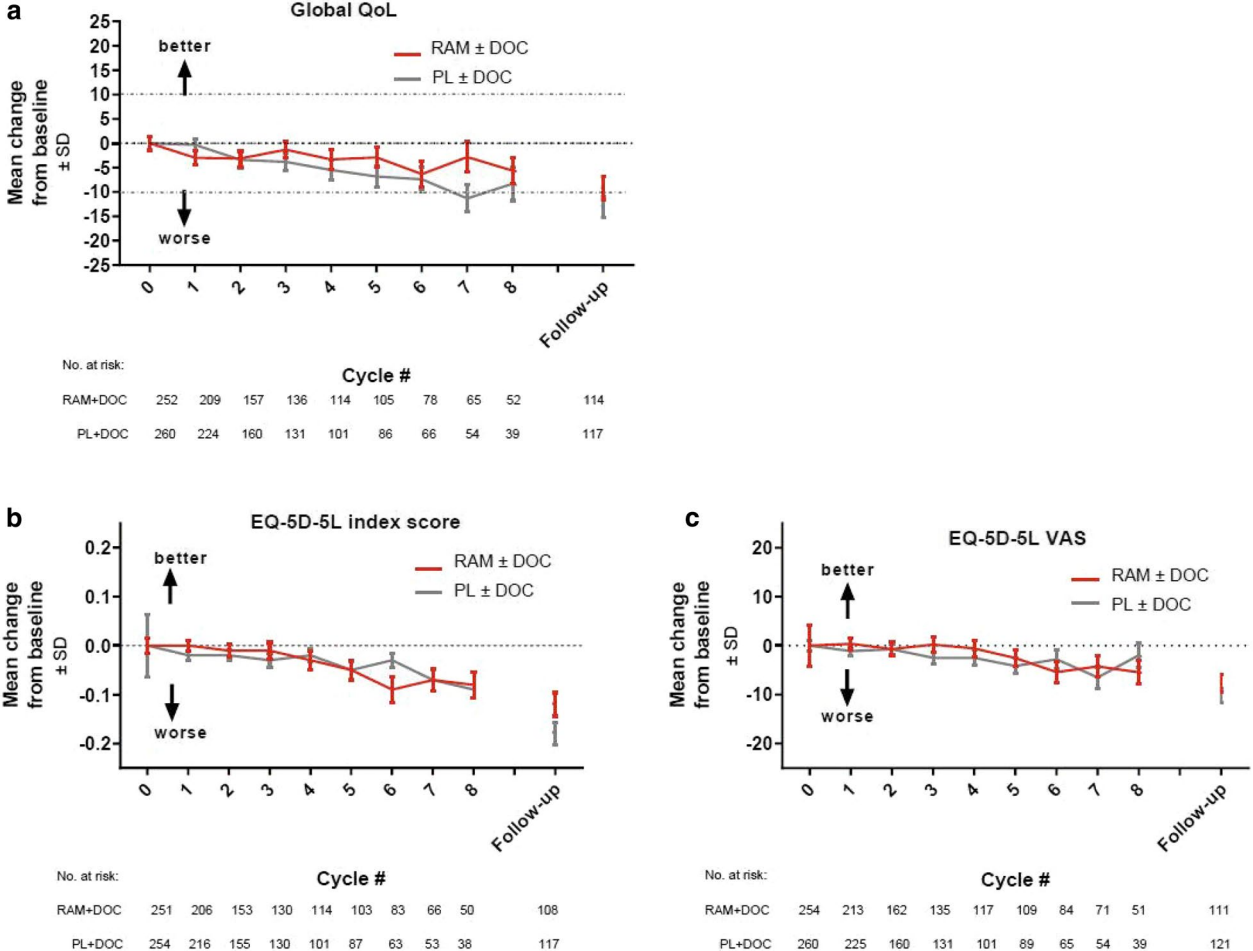
Mean change from baseline in the mean scores of the EORTC QLQ-C30 global QoL scale (**a**), EQ-5D-5L index (**b**) and visual analogue scale scores (**c**). *DOC* docetaxel, *EORTC QLQ-C30* European Organization for Research and Treatment of Cancer Quality of Life Questionnaire C30, *EQ-5D-5L* EuroQoL five-dimensions, *RAM* ramucirumab, *SD* standard deviation, *VAS* visual analogue scale

When considering high-level assessment of QoL and health status, no clear differences were observed between the treatment arms for changes in mean scores from baseline of the QLQ-C30 global QoL scale, EQ-5D-5L index, and EQ-5D-5L VAS (Fig. 2a–c). Although scores within arms worsened over time, the mean changes for on-treatment assessments were small. Similar patterns of no differences between arms in changes from baseline were observed for the other QoL scales, with the exception of pain (Additional file 1: Figure S1). For the functional scales, worsened scores over time were observed for physical and role functioning. For symptom scales, fatigue, dyspnea, and diarrhea had the greatest worsening over time. In the case of pain, improved scores were observed in the ramucirumab arm over the first five cycles, while scores worsened in the placebo arm. Additional file 1: Figure S2 summarizes the distribution of EQ-5D-5L responses over time. At baseline, the highest levels of impairment (i.e., moderate or more severe) were reported for pain/discomfort. In general, the distributions of responses for all dimensions were similar over time for on-treatment assessments (Additional file 1: Figure S2A-E). For all scales, change from baseline was worst at the post-discontinuation follow-up assessment. Thus, in general, findings of the QLQ-C30 and the EQ-5D-5L were consistent.

### Time to sustained deterioration

**Fig. 3:**
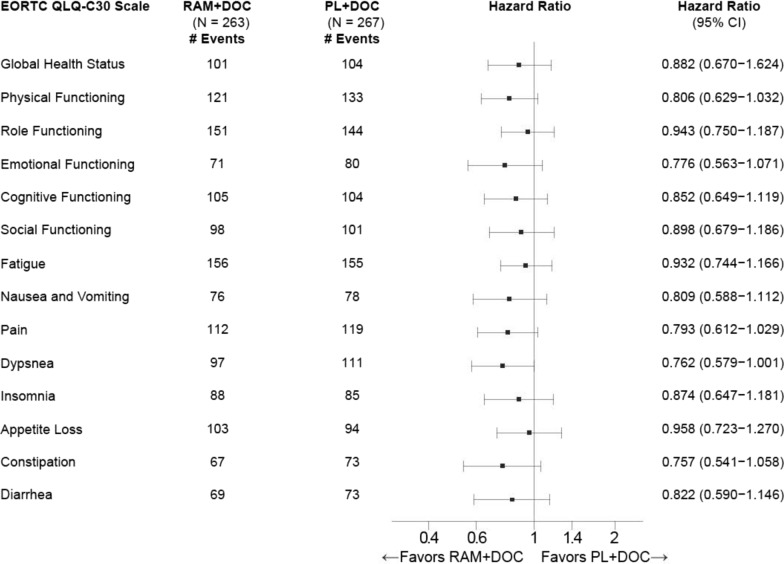
Time to sustained deterioration of QLQ-C30 scales. *CI* confidence interval; *DOC* docetaxel, *EORTC QLQ-C30* European Organization for Research and Treatment of Cancer Quality of Life Questionnaire C30, *N* number of patients, *HR* hazard ratio, *PL* placebo, *QoL* quality of life; *RAM* ramucirumab. Forest plot of time to sustained deterioration, HRs (unstratified) for each functional and symptom scale are depicted. HRs were estimated using a Cox regression model. 2-sided p-values were based on normal approximation

Results for TtD of QLQ-C30 scales are summarized in Fig. 3. The HR was < 1 for all scales (range 0.76–0.96), indicating a trend towards longer TtD in the ramucirumab arm. Kaplan–Meier figures are presented for those scales with ˂65% censoring of events (Additional file 1: Figure S3A-I). Median TtD for global QoL was 6.9 months (95% CI 4.2 months—8.9 months) vs. 4.6 months (95% CI 3.5 months—5.5 months) (HR 0.88, 95% CI 0.7–1.2).

### Rates of improved or stable scores

**Fig. 4:**
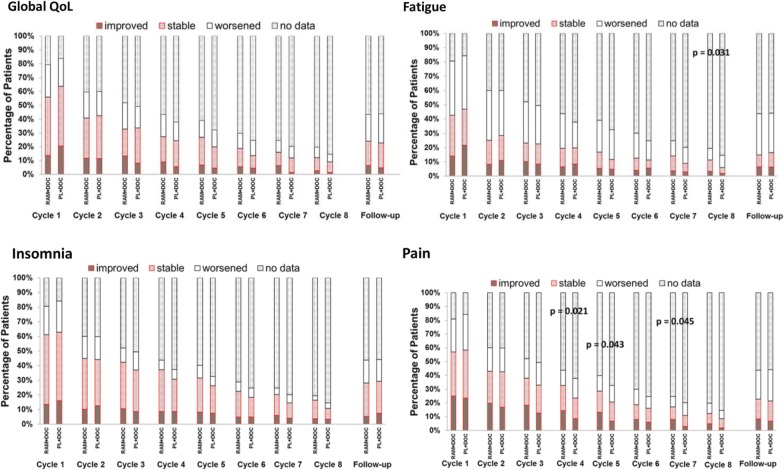
Proportion of patients in ITT population with improved, stable or worsened QoL of the scales with greatest impairment at baseline. *QoL* quality of life, *ITT* intention-to-treat. For each scale at each postbaseline assessment, proportion of patients with improved or stable scores was compared using the Fisher’s exact test

The proportion of patients whose QoL scores improved, remained stable, or deteriorated from baseline at each postbaseline assessment are shown in Fig. 4, for the scales with the greatest impairment at baseline; Additional file 1: Figure S4 depicts these results for all other scales. For each scale, rates of improved/stable were similar between treatment arms in early cycles. Of note, a higher proportion of patients in the ramucirumab arm had either improved or stable pain scores at cycles 4, 5, and 7 compared to the placebo arm (Fig. 4d). Indeed, across all cycles, the pain scale, relative to the other scales (Fig. 3a and Additional file 1: S4), exhibited consistency with respect to having the greatest proportion of patients with improvement in pain within the ramucirumab arm.

### Exploratory analyses

**Fig. 5:**
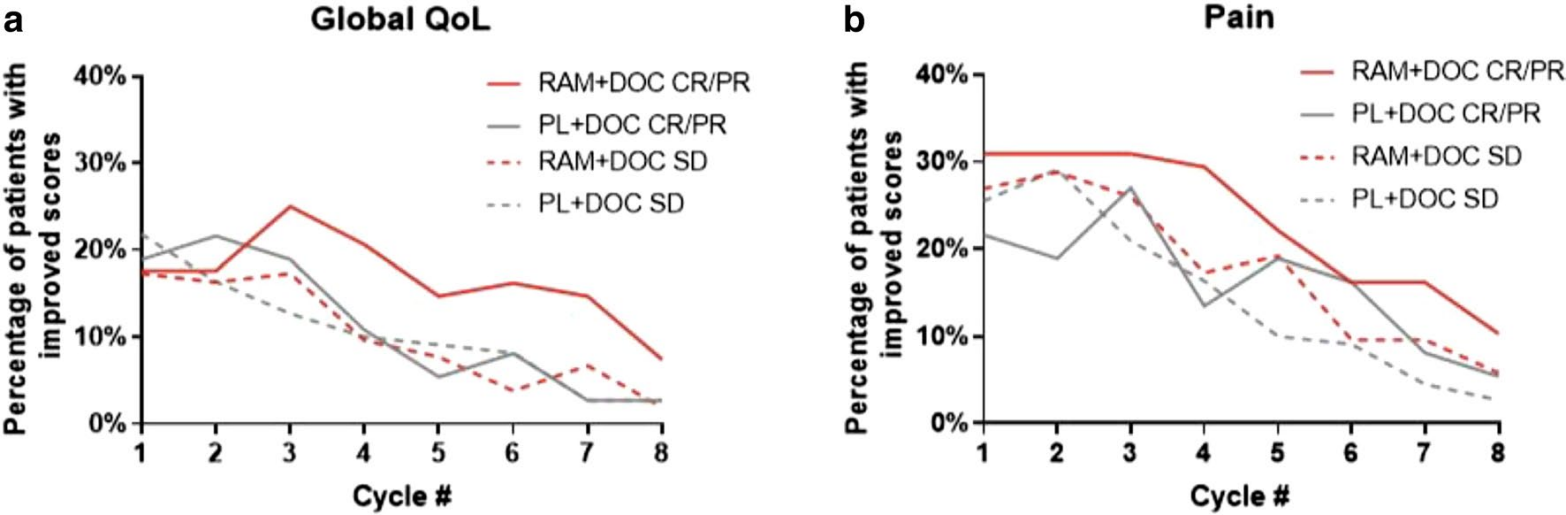
Proportion of patients with improvement in global QoL (**a**) and pain (**b**) scales among those patients with BOR of CR/PR or SD. *BOR* best overall response, *CR* complete response, *DOC* docetaxel, *n* number of patients, *PL* placebo, *PR* partial response, *QoL* quality of life; *RAM* ramucirumab, *SD* stable disease. For each scale at each postbaseline assessment, proportion of patients with improved or stable scores between treatment and placebo arms was compared using the Fisher’s exact test. PR/CR as BOR: RAM + DOC (n = 68) and PL + DOC (n = 37). SD as BOR: RAM + DOC (n = 104) and PL + DOC (n = 110)

Considering all prespecified analyses, the consistency of the pain results prompted an exploratory analysis evaluating the association with best overall response to treatment (Fig. 5). Because the global QoL scale provides the most holistic assessment, it was similarly evaluated. During the first four cycles, the range of patients achieving complete response (CR)/partial response (PR) with improved global QoL was 17.6–25.0% for ramucirumab and 10.8–21.6% for placebo; the range for patients with stable disease (SD) was 9.6–17.3% for ramucirumab and 10.0–21.8% for placebo (Fig. 5a). During the first four cycles, the range of patients achieving CR/PR with improved pain was 29.4–30.9% for ramucirumab and 13.5–27.0% for placebo; the range for patients with SD was 17.3–28.8% for ramucirumab and 16.4–29.1% for placebo (Fig. 5b).

In general, a higher proportion of patients who achieved CR/PR had improved pain scores in the ramucirumab arm versus the placebo arm. No apparent difference in pain palliation was seen between arms for patients who achieved SD. Similar results were observed for improved global QoL between ramucirumab versus placebo arms in patients who achieved CR/PR and SD (Fig. 5).

## Discussion

QoL is of utmost importance when considering therapeutic options for patients in the palliative setting [4, 18]. There can be concerns when a second agent is added to chemotherapy if gains in efficacy are also accompanied by increased toxicity [19], such as in the RANGE study where there were numerically higher rates of any-grade diarrhea, decreased appetite, nausea, and stomatitis in the ramucirumab plus docetaxel arm compared with the placebo plus docetaxel arm [3]. Thus, evaluating the effects on QoL through PROs when ramucirumab plus docetaxel was compared to placebo plus docetaxel was a key secondary endpoint in the RANGE trial. While on therapy, patients in the placebo plus docetaxel arm generally maintained global QoL and health status. Results were similar for the ramucirumab plus docetaxel arm, suggesting no detrimental impact. Worsening at the 30-day follow-up visit in both treatment arms was potentially associated with the negative impact of disease progression, which was the most common reason for treatment discontinuation. The longer PFS was consistent with trend for longer TtD in global QoL in the ramucirumab plus docetaxel arm. These findings may be helpful when considering the value of delaying disease progression and the balance of benefit and risk of combination therapy from the patient perspective.

In our study, all of the more specific aspects of QoL were at least maintained in the ramucirumab plus docetaxel arm relative to the placebo plus docetaxel arm. The strongest trend of delay in deterioration among the functional scales favoring ramucirumab plus docetaxel was for physical functioning which addresses mobility and self-care. Fatigue and insomnia were two of the most prominent symptoms reported by patients at baseline. Changes in fatigue were similar between arms, with patients more likely to report worsening. Insomnia scores generally remained similar both between and within arms.

Of all of the dimensions assessed by the QLQ-C30 and the EQ-5D-5L, pain may be the one most closely associated with disease symptoms and treatment efficacy, with less confounding by toxicity of treatment [20–22]. At baseline, the mean score for pain from the QLQ-C30 indicated high levels of pain and < 30% of patients reported no pain or discomfort on the EQ-5D-5L. All analyses of pain from the QLQ-C30 suggested a differential effect between arms. In the descriptive summaries, mean scores generally improved for the ramucirumab plus docetaxel arm, but worsened for the placebo plus docetaxel arm. At later cycles, more patients in the ramucirumab plus docetaxel arm reported improved or stable pain scores. The HR for TtD was 0.79 (95% CI 0.61, 1.0). In the exploratory analysis conducted, patients treated with ramucirumab plus docetaxel, who achieved a complete or partial response, often reported improvement in pain. The observed relative benefit in pain palliation may be associated with the greater extent of tumor shrinkage among responders and longer duration of response seen in the ramucirumab plus docetaxel arm. The association of symptom palliation with tumor response in other populations has been previously reported [23, 24].

Recent phase 3 studies of immune checkpoint inhibitors in similar study populations also assessed QoL [25, 26]. Although trial designs differed, the control arms were single-agent chemotherapy, and QoL was assessed with the QLQ-C30. However, different analysis approaches limit comparisons across the studies. In general, chemotherapy-based regimens were associated with within-arm worsening in global QoL over time and checkpoint inhibitors were associated with no within-arm improvement or worsening. In KEYNOTE-045, the HR for time to first deterioration in global QoL was0.72, indicating a longer time to first deterioration for pembrolizumab compared to chemotherapy [25]. In IMvigor211, the HR for time to sustained deterioration in global QoL was not reported, but median values were the same for atezoluzimab and chemotherapy [26].The HR for time to sustained deterioration in global QoL in RANGE was 0.887, trending for a longer time to deterioration for ramucirumab plus docetaxel compared to chemotherapy (docetaxel). In RANGE, the addition of ramucirumab to chemotherapy did not further worsen QoL but demonstrated within-arm improvements in pain scores. Both RANGE and KEYNOTE-045 [25] observed a worsening of QoL associated with disease progression. This supports our findings that disease-related symptoms may have a more prominent effect on QoL in this setting.

This study used robust analytical methods. The analysis was prespecified and completion rate of questionnaires was high during the study. The ramucirumab and placebo groups were similar at baseline in PROs and patient and disease characteristics. This study showed no deterioration of QoL with addition of ramucirumab, with consistency observed across QLQ-C30 global QoL scale, EQ-5D-5L index, and VAS scores. The completion rate at the post-discontinuation follow-up visit in our study was comparable to other studies in advanced cancer [27, 28].In our study slightly more than half of patients provided post-discontinuation data, limiting the characterization of QoL and health status for that time period.

Limitations in the study include PRO instruments, patient characteristics, and incomplete follow-up data. Although cancer-specific and the most widely used in aUC trials, the QLQ-C30 is not specific to UC. No validated tumor-specific module to supplement the QLQ-C30 is available for use to assess additional concerns of aUC patients [29, 30]. With the baseline eligibility criterion of ECOG performance status of zero or one, there is less opportunity for patients to report improvements in PROs. As is common in advanced cancer trials, early discontinuation of patients results in non-random missing data; therefore, we attempted to minimize the impact by conducting cycle-by-cycle analysis to explore data trend instead of focusing on only certain cycle as a snapshot. We also made the general assumption that patients who discontinued therapy likely have worsened QoL, as might be expected with disease progression which was most common reason for study discontinuation. While PRO completion rates were lower at follow-up, characteristics and outcomes of patients who provided follow-up data were similar to those who did not. However, despite these limitations, trends in differences between arms were observed that were consistent with other clinical outcomes.

As the term itself suggests, PROs are reported by patients themselves without any interpretation from a physician or anyone else. PROs can be used to support evaluation of response to treatment and to increase clinician-patient engagement [31]. Integration of PROs to the care demonstrated a survival benefit in patients with cancer compared with patients undergoing usual care (median 31.2 months vs. median 26.0 months, *p* = 0.03) [6]. In addition, PROs provide patients with a mechanism to self-report symptoms, minimize a decline in QoL, reduce hospitalization and emergency room admissions, and prolonged time on chemotherapy [32]. As newer agents become available for aUC, PRO/QoL data should be provided to help clinicians make informed treatment decisions [33–35].

## Conclusions

In summary, QoL outcomes of the phase 3 RANGE trial presented here provided additional insights regarding no negative impact of ramucirumab plus docetaxel on QoL compared with placebo plus docetaxel. The association of pain palliation with tumor response and disease control observed in this study may be used when considering therapeutic choices for advanced UC.

## Supplementary information

**Additional file 1.** **Figure S1.** Change from baseline in QLQ-C30 scales (except Global QoL). DOC = docetaxel; PL = placebo; QLQ-C30 = Cancer Quality of Life Questionnaire C30; RAM = ramucirumab; SEM = standard error of the mean. **Figure S2.** Distributions of dimension responses for EQ-5D-5L (patients who provided data) DOC = docetaxel; EQ-5D-5L = EQ-5D-5L = EuroQoL five-dimensions; n = number of patients; PL = placebo; RAM = ramucirumab. **Figure S3.** Kaplan-Meier TtD plots of QLQ-C30 scales with less than 65% censoring CI = confidence interval; HR = hazard ratio. **Figure S4.** Proportion of patients with improved, stable, or worsened QLQ-C30 scales (except global QoL, fatigue, pain, insomnia) DOC = docetaxel; PL = placebo; QLQ-C30 = Cancer Quality of Life Questionnaire C30; QoL = quality of life; RAM = ramucirumab.

## Data Availability

Lilly provides access to all individual participant data collected during the trial, after anonymization, with the exception of pharmacokinetic or genetic data. Data are available to request 6 months after the indication studied has been approved in the US and EU and after primary publication acceptance, whichever is later. No expiration date of data requests is currently set once they are made available. Access is provided after a proposal has been approved by an independent review committee identified for this purpose and after receipt of a signed data sharing agreement. Data and documents, including the study protocol, statistical analysis plan, clinical study report, and blank or annotated case report forms, will be provided in a secure data-sharing environment for up to 2 years per proposal. The data that support the findings of this study are available from Clinicalstudydatarequest.com, but restrictions apply to the availability of these data, which were used under license for the current study, and so are not publicly available. Data are however available from the authors upon reasonable request and with permission of www.clinicalstudydatarequest.com. For details on submitting a request, see the instructions provided at www.clinicalstudydatarequest.com under “How it works”.

## Abbreviations

aUC: Advanced urothelial carcinoma
CI: Confidence interval
ECOG: Eastern Cooperative Oncology Group
EORTC QLQ-C30: European Organization for Research and Treatment of Cancer Quality of Life Questionnaire C30
EQ-5D-5L: EuroQoL five-dimensions, 5 level
HR: Hazard ratio
IV: Intravenously
OS: Overall survival
PRO: Patient-reported outcome
QoL: Quality of life
VEGF: Vascular endothelial growth factor
VEGFR: Vascular endothelial growth factor receptor

## References

[1] Cumberbatch K, He T, Thorogood Z, Gartrell BA (2017). Emerging drugs for urothelial (bladder) cancer. Expert Opin Emerg Drugs.

[2] Petrylak DP, de Wit R, Chi KN, Drakaki A, Sternberg CN, Nishiyama H, Castellano D, Hussain S, Flechon A, Bamias A (2017). Ramucirumab plus docetaxel versus placebo plus docetaxel in patients with locally advanced or metastatic urothelial carcinoma after platinum-based therapy (RANGE): a randomised, double-blind, phase 3 trial. Lancet.

[3] Petrylak DP, de Wit R, Chi KN, Drakaki A, Sternberg CN, Nishiyama H, Castellano D, Hussain SA, Flechon A, Bamias A (2020). Ramucirumab plus docetaxel versus placebo plus docetaxel in patients with locally advanced or metastatic urothelial carcinoma after platinum-based therapy (RANGE): overall survival and updated results of a randomised, double-blind, phase 3 trial. Lancet Oncol.

[4] Howie L, Peppercorn J (2013). Early palliative care in cancer treatment: rationale, evidence and clinical implications. Ther Adv Med Oncol.

[5] Orom H, Biddle C, Underwood W, Nelson CJ, Homish DL (2016). What is a "good" treatment decision? decisional control, knowledge, treatment decision making, and quality of life in men with clinically localized prostate cancer. Med Decis Making.

[6] Basch E, Deal AM, Dueck AC, Scher HI, Kris MG, Hudis C, Schrag D (2017). Overall survival results of a trial assessing patient-reported outcomes for symptom monitoring during routine cancer treatment. JAMA.

[7] Sato K, Sasaki R, Ogura Y, Shimoda N, Togashi H, Terada K, Sugiyama T, Kakinuma H, Ogawa O, Kato T (1998). Expression of vascular endothelial growth factor gene and its receptor (flt-1) gene in urinary bladder cancer. Tohoku J Exp Med.

[8] Xia G, Kumar SR, Hawes D, Cai J, Hassanieh L, Groshen S, Zhu S, Masood R, Quinn DI, Broek D (2006). Expression and significance of vascular endothelial growth factor receptor 2 in bladder cancer. J Urol.

[9] Lu D, Shen J, Vil MD, Zhang H, Jimenez X, Bohlen P, Witte L, Zhu Z (2003). Tailoring in vitro selection for a picomolar affinity human antibody directed against vascular endothelial growth factor receptor 2 for enhanced neutralizing activity. J Biol Chem.

[10] Poole RM, Vaidya A (2014). Ramucirumab: first global approval. Drugs.

[11] Aragon-Ching JB, Trump DL (2017). Targeted therapies in the treatment of urothelial cancers. Urol Oncol.

[12] Mazzola CR, Chin J (2015). Targeting the VEGF pathway in metastatic bladder cancer. Expert Opin Investig Drugs.

[13] Eisenhauer EA, Therasse P, Bogaerts J, Schwartz LH, Sargent D, Ford R, Dancey J, Arbuck S, Gwyther S, Mooney M (2009). New response evaluation criteria in solid tumours: revised RECIST guideline (version 1.1). Eur J Cancer.

[14] Aaronson NK, Ahmedzai S, Bergman B, Bullinger M, Cull A, Duez NJ, Filiberti A, Flechtner H, Fleishman SB, de Haes JC (1993). The European Organization for Research and Treatment of Cancer QLQ-C30: a quality-of-life instrument for use in international clinical trials in oncology. J Natl Cancer Inst.

[15] Herdman M, Gudex C, Lloyd A, Janssen M, Kind P, Parkin D, Bonsel G, Badia X (2011). Development and preliminary testing of the new five-level version of EQ-5D (EQ-5D-5L). Qual Life Res.

[16] Osoba D, Rodrigues G, Myles J, Zee B, Pater J (1998). Interpreting the significance of changes in health-related quality-of-life scores. J Clin Oncol.

[17] Devlin NJ, Shah KK, Feng Y, Mulhern B, van Hout B (2018). Valuing health-related quality of life: An EQ-5D-5L value set for England. Health Econ.

[18] Kaasa S, Loge JH, Aapro M, Albreht T, Anderson R, Bruera E, Brunelli C, Caraceni A, Cervantes A, Currow DC (2018). Integration of oncology and palliative care: a Lancet Oncology Commission. Lancet Oncol.

[19] Yafi FA, North S, Kassouf W (2011). First- and second-line therapy for metastatic urothelial carcinoma of the bladder. Curr Oncol.

[20] Taarnhoj GA, Johansen C, Pappot H (2019). Quality of life in bladder cancer patients receiving medical oncological treatment; a systematic review of the literature. Health Qual Life Outcomes.

[21] Serpentini S, Del Bianco P, Chirico A, Merluzzi TV, Martino R, Lucidi F, De Salvo GL, Trentin L, Capovilla E (2019). Self-efficacy for coping: utility of the Cancer behavior inventory (Italian) for use in palliative care. BMC Palliat Care.

[22] Huang W, Yang J, Liu Y, Liu C, Zhang X, Fu W, Shi L, Liu G (2018). Assessing health-related quality of life of patients with colorectal cancer using EQ-5D-5L: a cross-sectional study in Heilongjiang of China. BMJ Open.

[23] Geels P, Eisenhauer E, Bezjak A, Zee B, Day A (2000). Palliative effect of chemotherapy: objective tumor response is associated with symptom improvement in patients with metastatic breast cancer. J Clin Oncol.

[24] Chau I, Fuchs CS, Ohtsu A, Barzi A, Liepa AM, Cui ZL, Hsu Y, Al-Batran SE (2019). Association of quality of life with disease characteristics and treatment outcomes in patients with advanced gastric cancer: Exploratory analysis of RAINBOW and REGARD phase III trials. Eur J Cancer.

[25] Vaughn DJ, Bellmunt J, Fradet Y, Lee JL, Fong L, Vogelzang NJ, Climent MA, Petrylak DP, Choueiri TK, Necchi A (2018). Health-related quality-of-life analysis from KEYNOTE-045: a phase III study of pembrolizumab versus chemotherapy for previously treated advanced urothelial cancer. J Clin Oncol.

[26] Powles T, Duran I, van der Heijden MS, Loriot Y, Vogelzang NJ, De Giorgi U, Oudard S, Retz MM, Castellano D, Bamias A (2018). Atezolizumab versus chemotherapy in patients with platinum-treated locally advanced or metastatic urothelial carcinoma (IMvigor211): a multicentre, open-label, phase 3 randomised controlled trial. Lancet.

[27] Atkins MB, Rini BI, Motzer RJ, Powles T, McDermott DF, Suarez C, Bracarda S, Stadler WM, Donskov F, Gurney H (2020). Patient-reported outcomes from the phase III Randomized IMmotion151 Trial: Atezolizumab + Bevacizumab versus sunitinib in treatment-naive metastatic renal cell carcinoma. Clin Cancer Res.

[28] Unger JM, Griffin K, Donaldson GW, Baranowski KM, Good MJ, Reburiano E, Hussain M, Monk PJ, Van Veldhuizen PJ, Carducci MA (2017). Patient-reported outcomes for patients with metastatic castration-resistant prostate cancer receiving docetaxel and Atrasentan versus docetaxel and placebo in a randomized phase III clinical trial (SWOG S0421). J Patient Rep Outcomes.

[29] EORTC. Quality of Life. Questionnairres. https://qol.eortc.org/questionnaires/. Accessed 7th May 2020.

[30] Mason SJ, Catto JWF, Downing A, Bottomley SE, Glaser AW, Wright P (2018). Evaluating patient-reported outcome measures (PROMs) for bladder cancer: a systematic review using the COnsensus-based Standards for the selection of health Measurement INstruments (COSMIN) checklist. BJU Int.

[31] Health USDo, Human Services FDACfDE, Research, Health USDo, Human Services FDACfBE, Research, Health USDo, Human Services FDACfD, Radiological H (2006). Guidance for industry: patient-reported outcome measures: use in medical product development to support labeling claims: draft guidance. Health Qual Life Outcomes.

[32] Basch E, Deal AM, Kris MG, Scher HI, Hudis CA, Sabbatini P, Rogak L, Bennett AV, Dueck AC, Atkinson TM (2016). Symptom monitoring with patient-reported outcomes during routine cancer treatment: a randomized controlled trial. J Clin Oncol.

[33] Loriot Y, Necchi A, Park SH, Garcia-Donas J, Huddart R, Burgess E, Fleming M, Rezazadeh A, Mellado B, Varlamov S (2019). Erdafitinib in locally advanced or metastatic urothelial carcinoma. N Engl J Med.

[34] Powles T, Park SH, Voog E, Caserta C, Valderamma BP, H G: Maintenance avelumab + best supportive care (BSC) versus BSC alone after platinum-based first-line (1L) chemotherapy in advanced urothelial carcinoma (UC): JAVELIN Bladder 100 phase III interim analysis**.** *J Clin Oncol 38: 2020 (suppl; abstr LBA1)* 2020.

[35] Rosenberg JE, O'Donnell PH, Balar AV, McGregor BA, Heath EI, Yu EY, Galsky MD, Hahn NM, Gartner EM, Pinelli JM (2019). Pivotal trial of enfortumab vedotin in urothelial carcinoma after platinum and anti-programmed death 1/programmed death ligand 1 therapy. J Clin Oncol.

