# Supplementary material for: Health-related quality of life in the randomized phase 3 study of ramucirumab plus docetaxel versus placebo plus docetaxel in platinum-refractory advanced urothelial carcinoma (RANGE)
Source: BMC Urol. 2020 Nov 7;20:181. doi: 10.1186/s12894-020-00752-w (PMC7648381; doi:10.1186/s12894-020-00752-w)
Supplement: Supplementary file 1 — Additional file 1. Figure S1. Change from baseline in QLQ-C30 scales (except Global QoL). DOC = docetaxel; PL = placebo; QLQ-C30 = Cancer Quality of Life Questionnaire C30; RAM = ramucirumab; SEM = standard error of the mean. Figure S2. Distributions of dimension responses for EQ-5D-5L (patients who provided data) DOC = docetaxel; EQ-5D-5L = EQ-5D-5L = EuroQoL five-dimensions; n = number of patients; PL = placebo; RAM = ramucirumab. Figure S3. Kaplan-Meier TtD plots of QLQ-C30 scales with less than 65% censoring CI = confidence interval; HR = hazard ratio. Figure S4. Proportion of patients with improved, stable, or worsened QLQ-C30 scales (except global QoL, fatigue, pain, insomnia) DOC = docetaxel; PL = placebo; QLQ-C30 = Cancer Quality of Life Questionnaire C30; QoL = quality of life; RAM = ramucirumab. [file 12894_2020_752_MOESM1_ESM.docx]

# **Supplementary Figures**

**Figure S1. Change from baseline of QLQ-C30 scales (except Global QoL)**

**
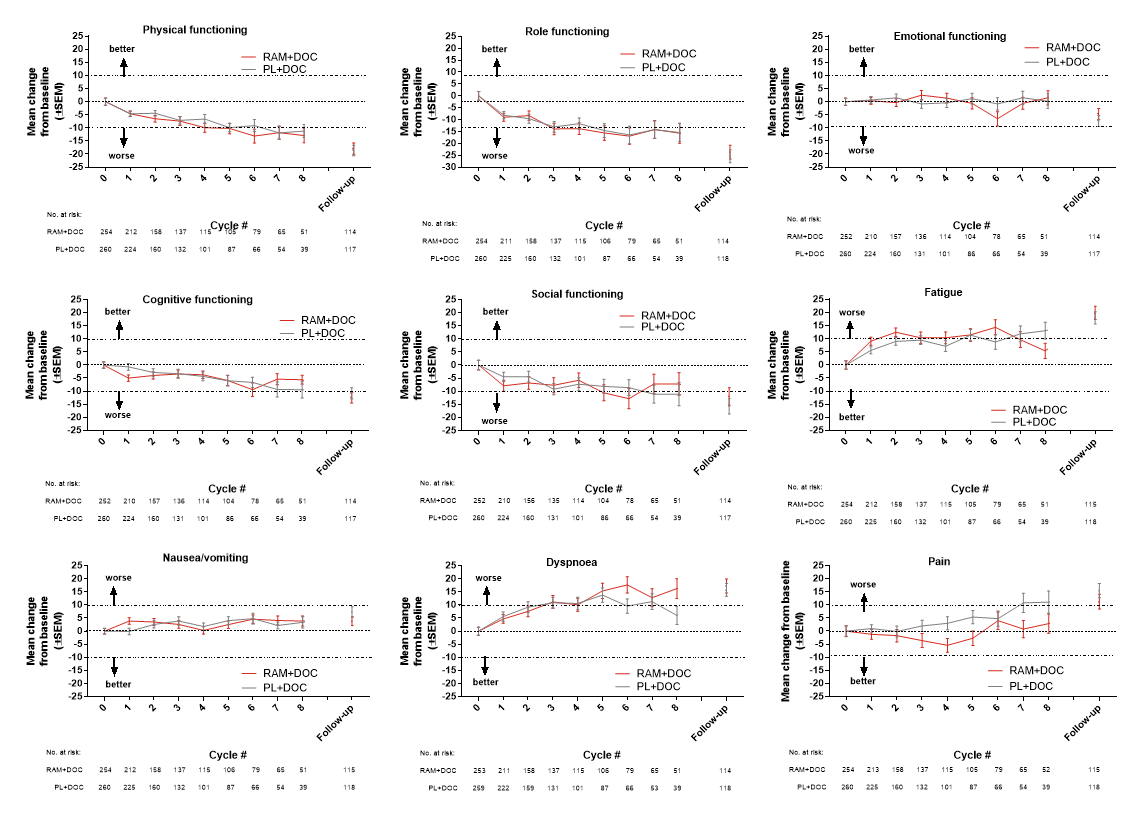
**

**Figure S1, continued**

DOC = docetaxel; PL = placebo; QLQ-C30 = Cancer Quality of Life Questionnaire C30; RAM = ramucirumab; SEM = standard error of the mean.

**Figure S2. Distributions of dimension responses for EQ-5D-5L (patients who provided data)**

1. **Mobility**


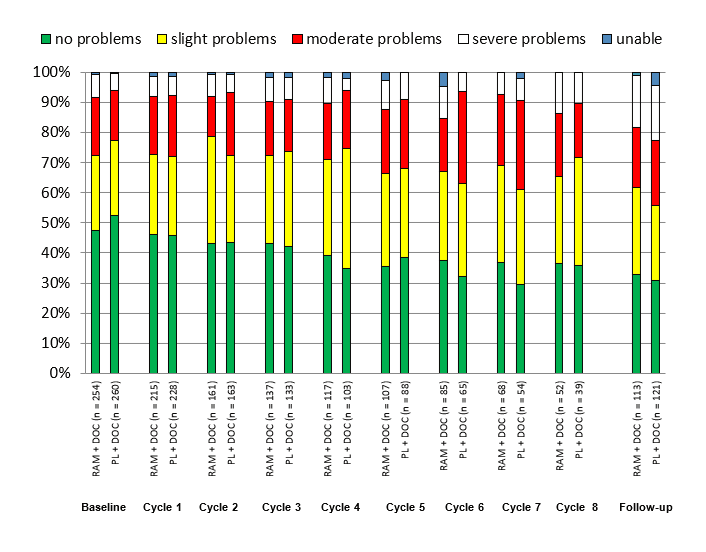


1. **Self-care**


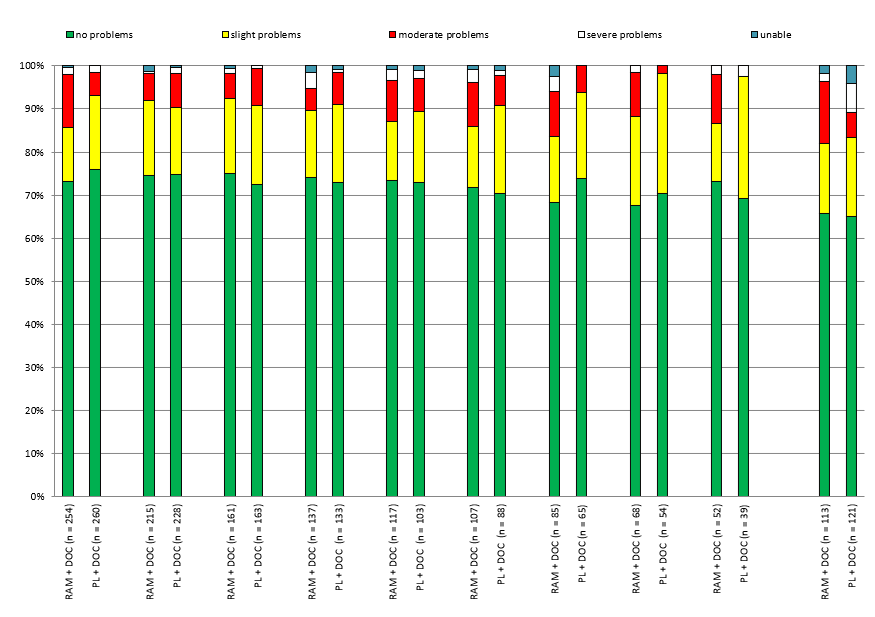


**Cycle 1**

**Cycle 2**

**Cycle 3**

**Cycle 4**

**Cycle 5**

**Cycle 6**

**Follow-up**

**Cycle 7**

**Cycle 8**

**Baseline**

1. **Usual activities**


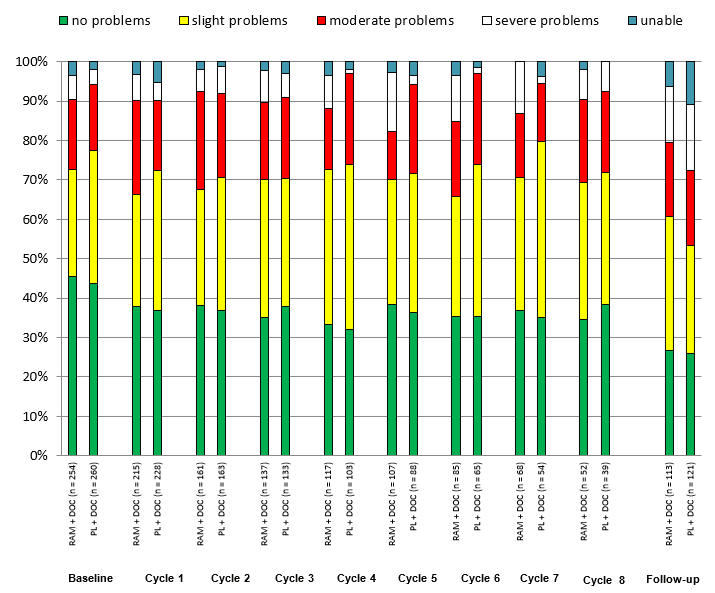


1. **Pain or discomfort**


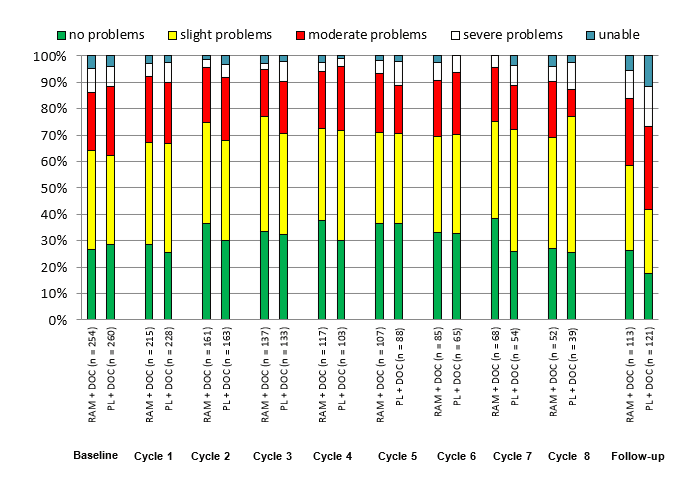


1. **Anxiety or depression**


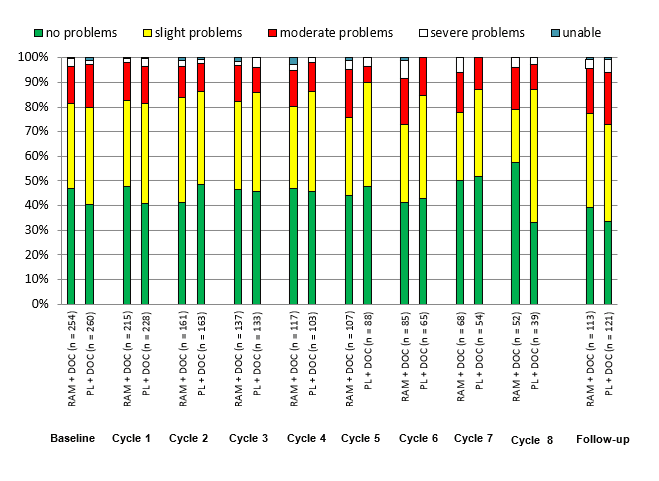


DOC = docetaxel; EQ-5D-5L = EQ-5D-5L = EuroQoL five-dimensions; n = number of patients; PL = placebo; RAM = ramucirumab.

**Figure S3. Kaplan-Meier TtD plots of QLQ-C30 scales with less than 65% censoring**

1. **Global QoL**

**
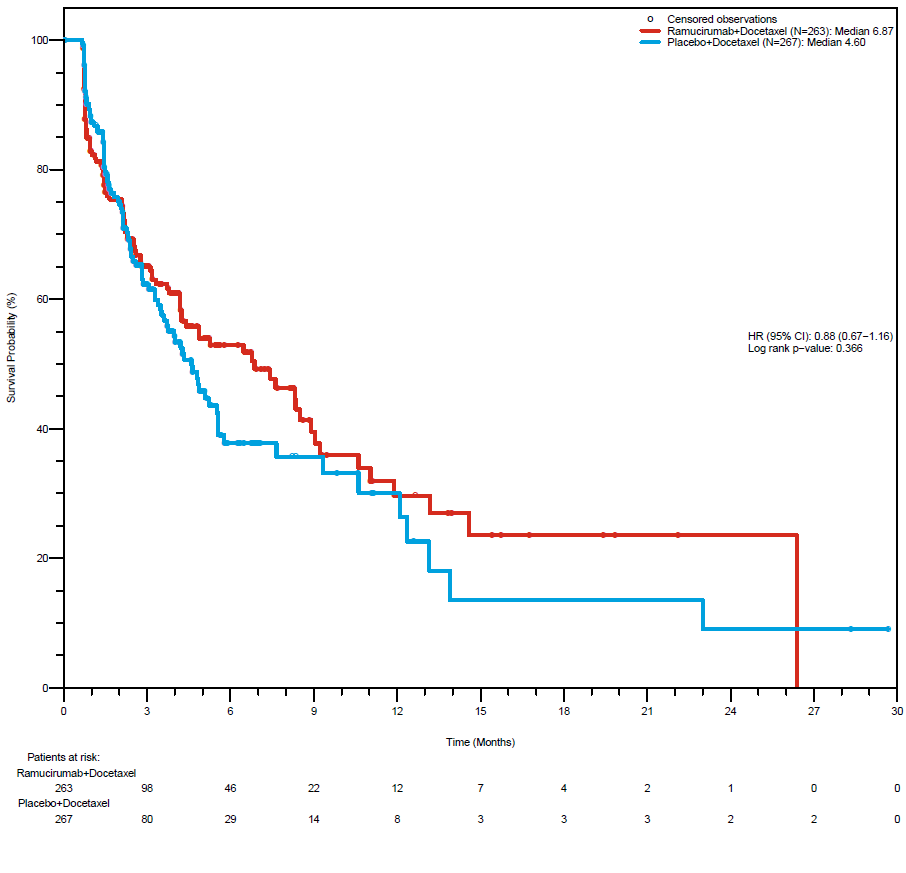
**

1. **Physical functioning**

**
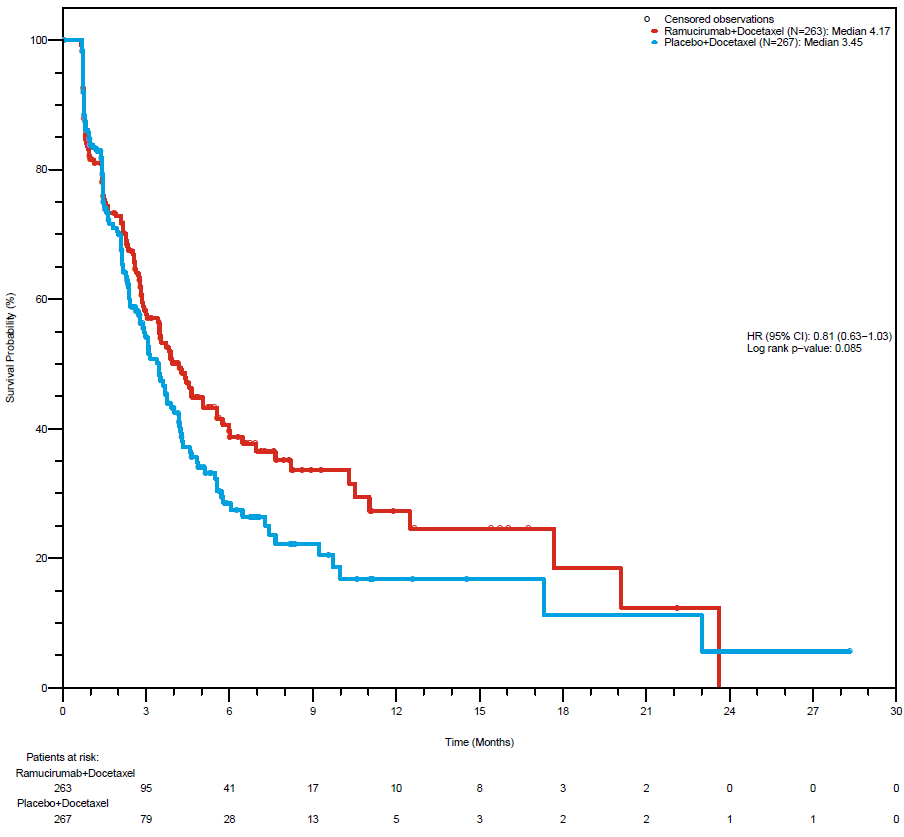
**

1. **Role functioning**

**
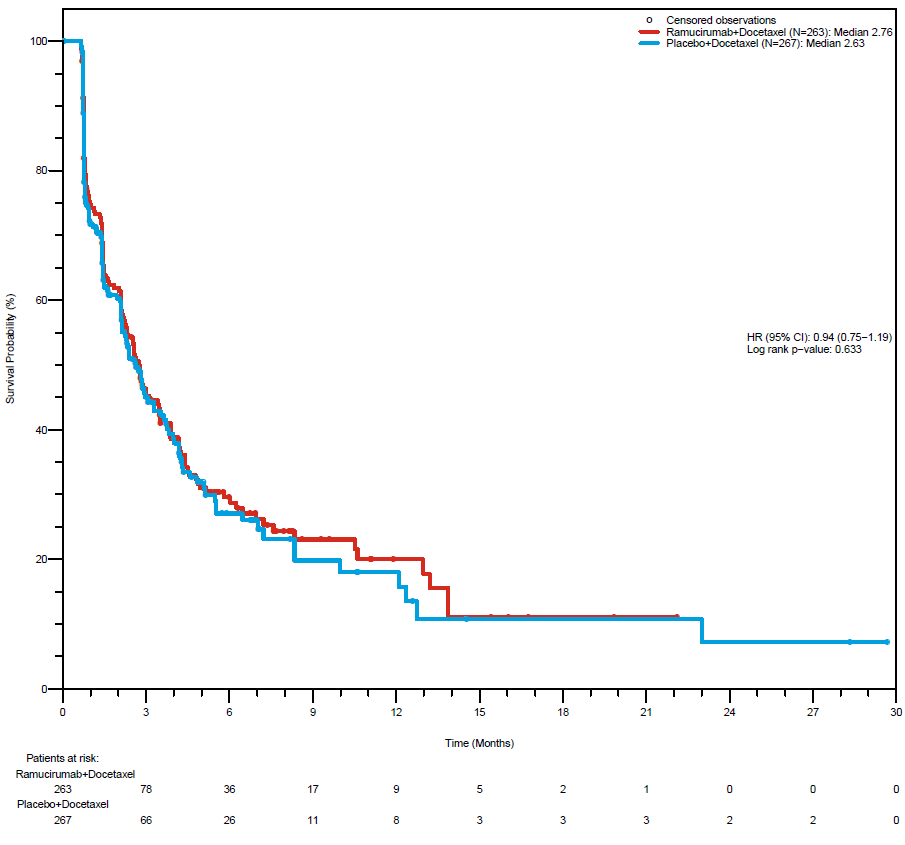
**

1. **Cognitive functioning**

**
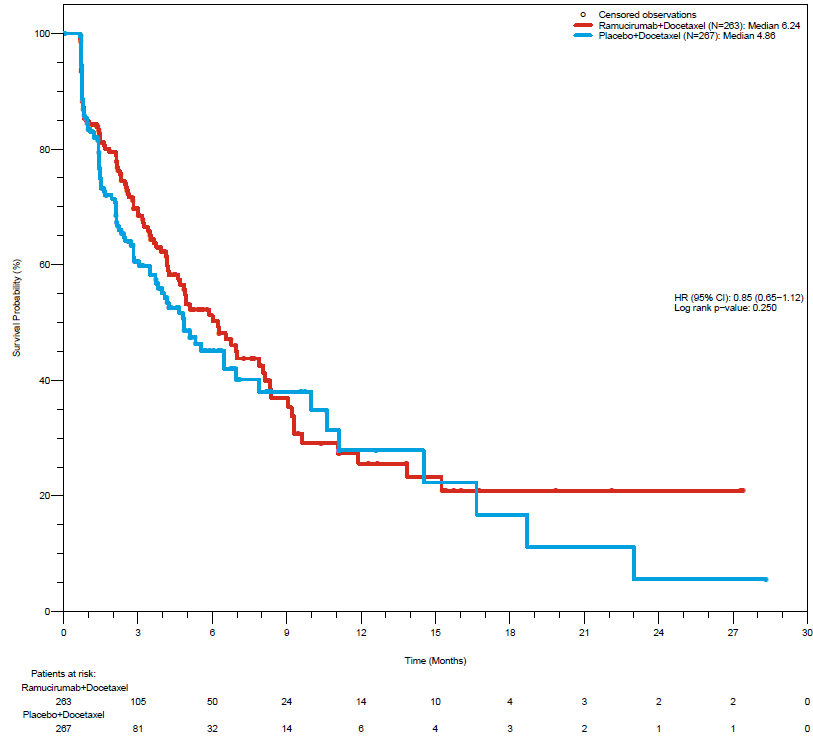
**

1. **Social functioning**

**
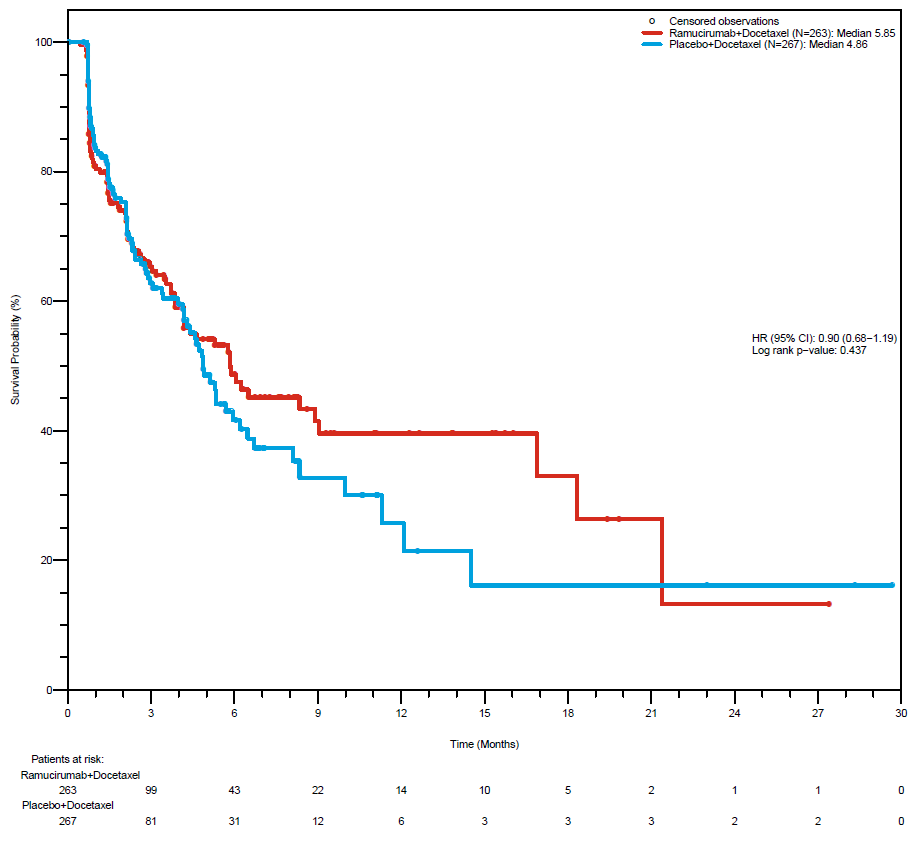
**

1. **Fatigue**

**
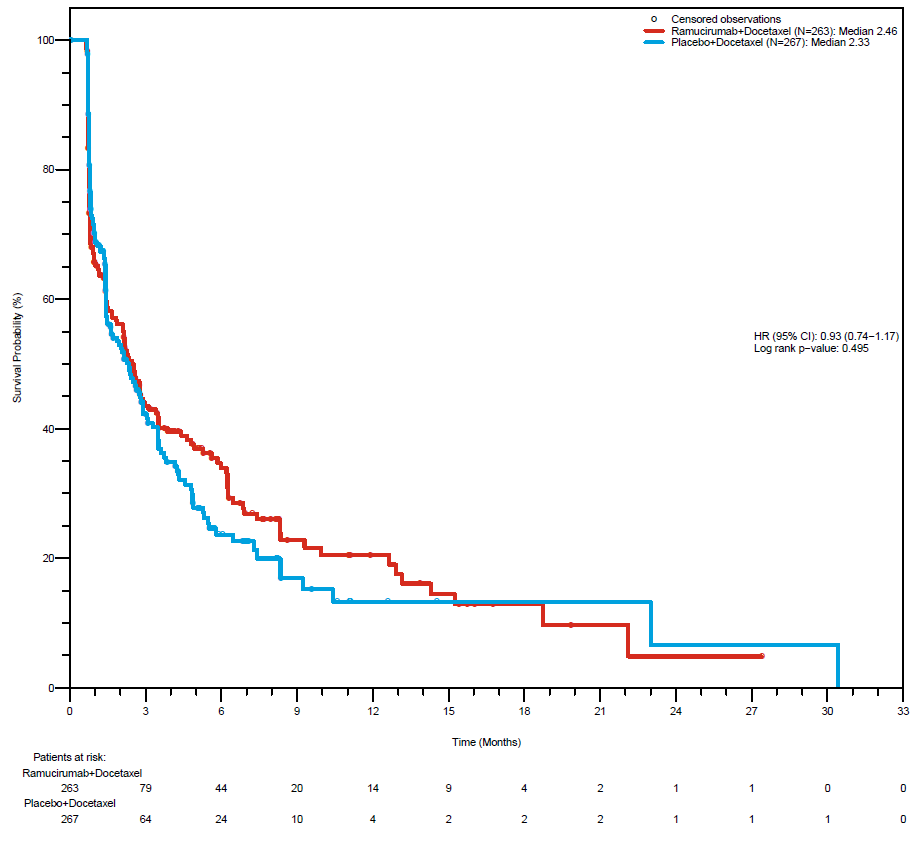
**

1. **Pain**

**
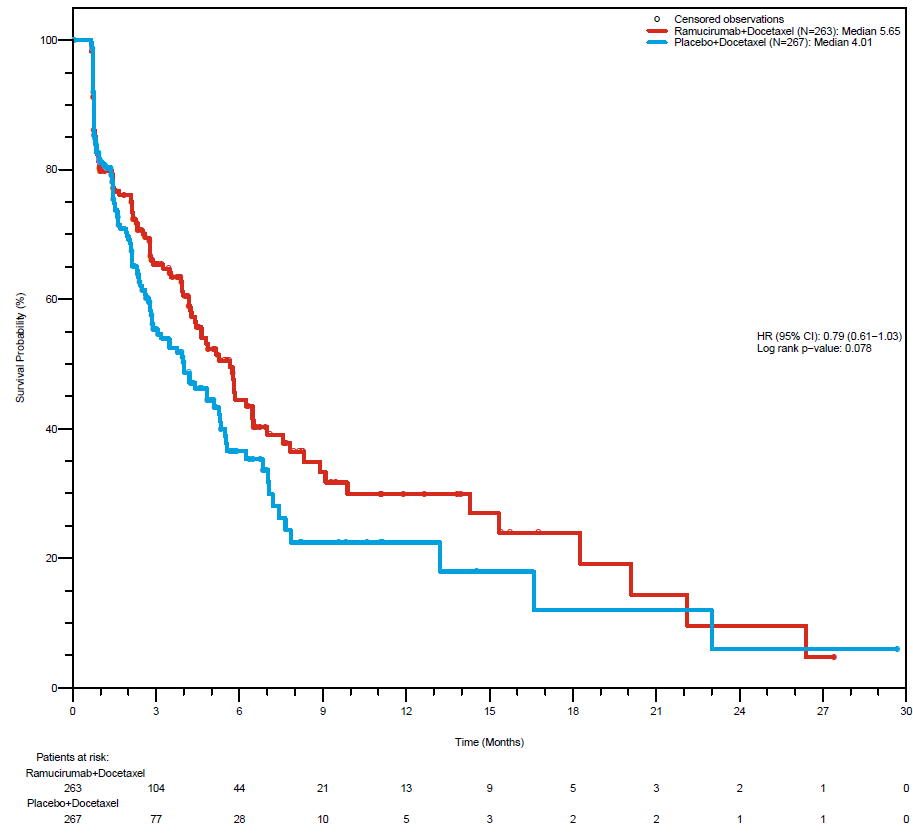
**

1. **Dyspnea**

**
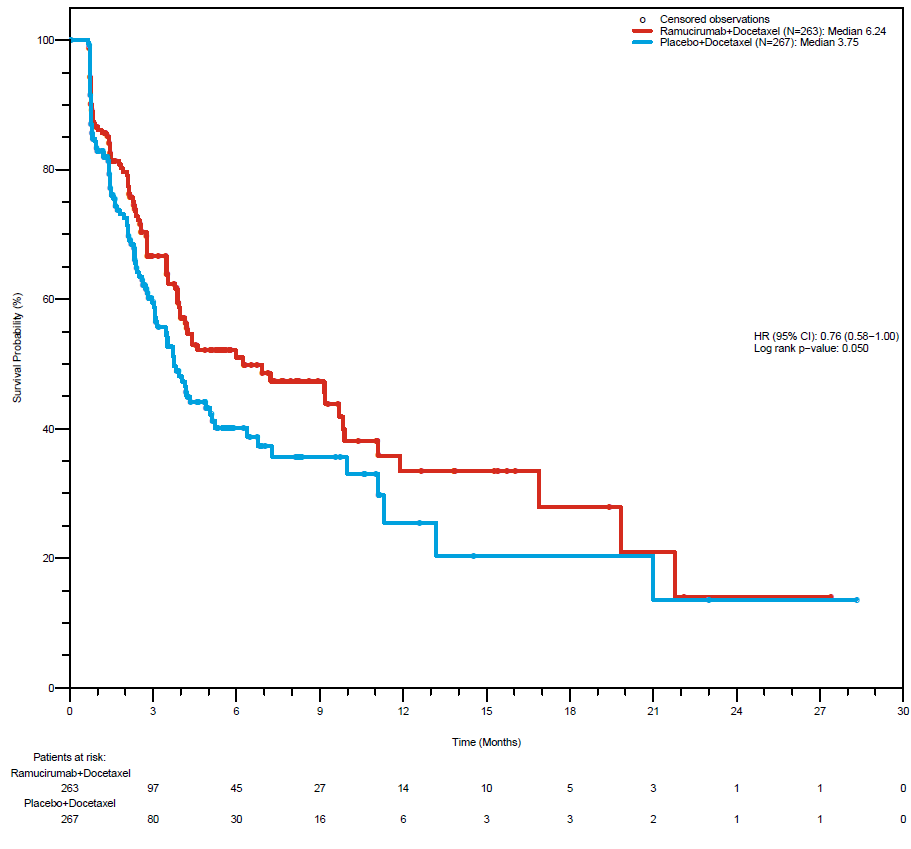
**

1. **Appetite loss**

**
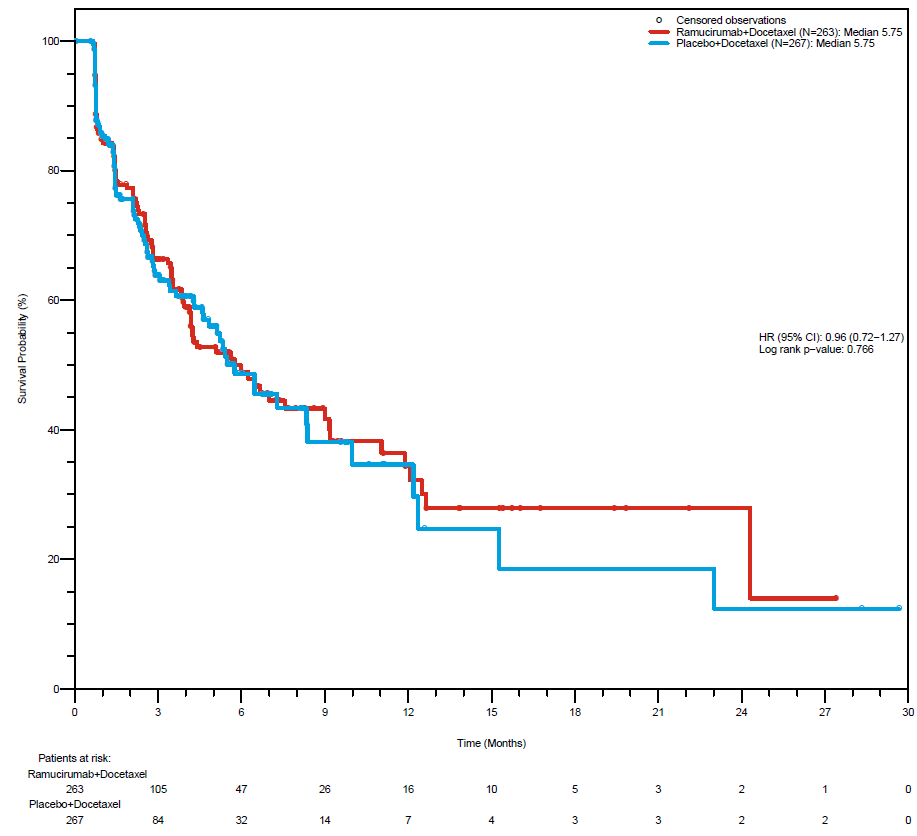
**

CI = confidence interval; HR = hazard ratio.

**Figure S4. Proportion of patients with improved, stable, or worsened QLQ-C30 scales (except global QoL, fatigue, pain, insomnia)**

**
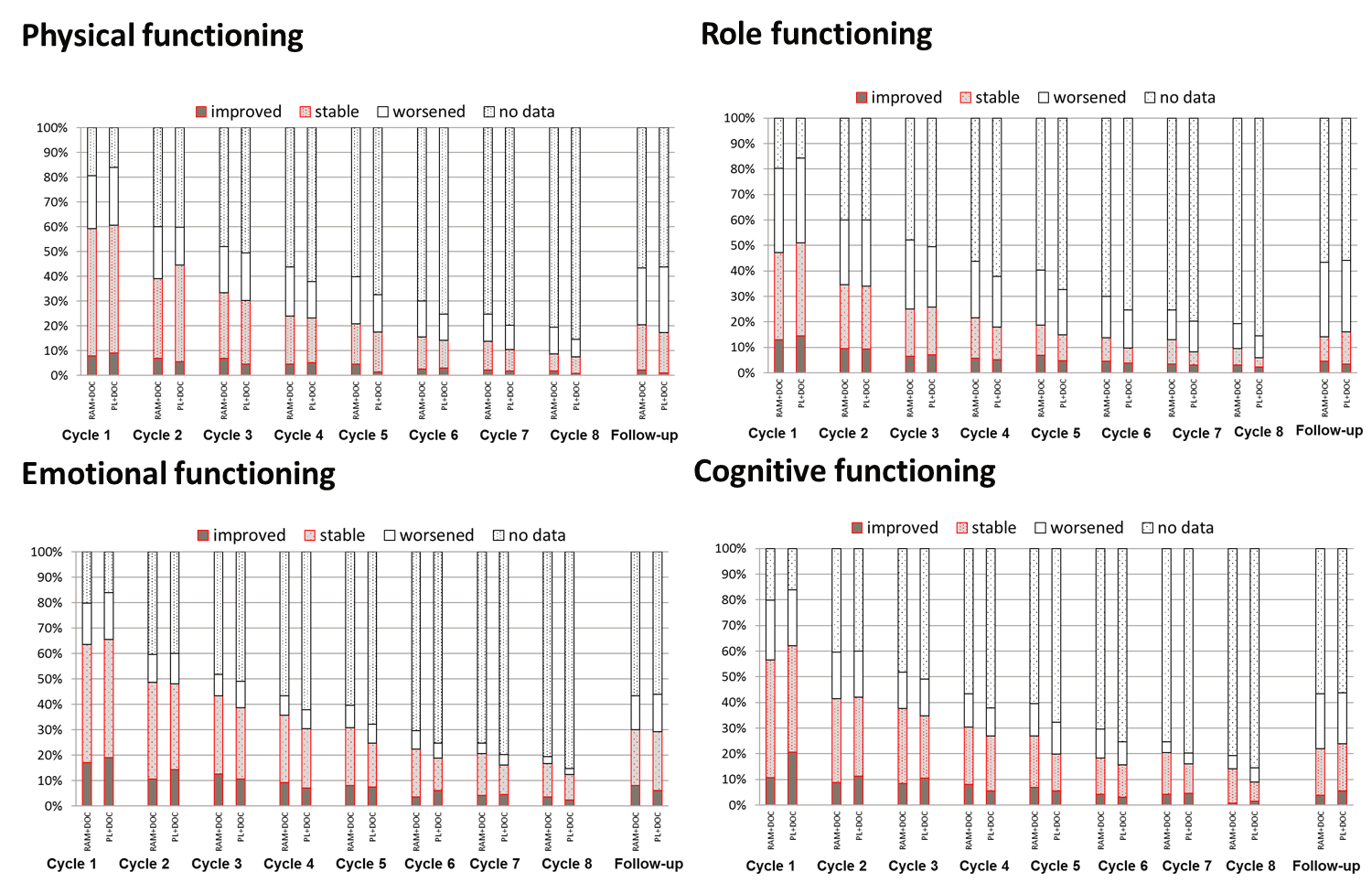
**

**Figure S4 continued**

**
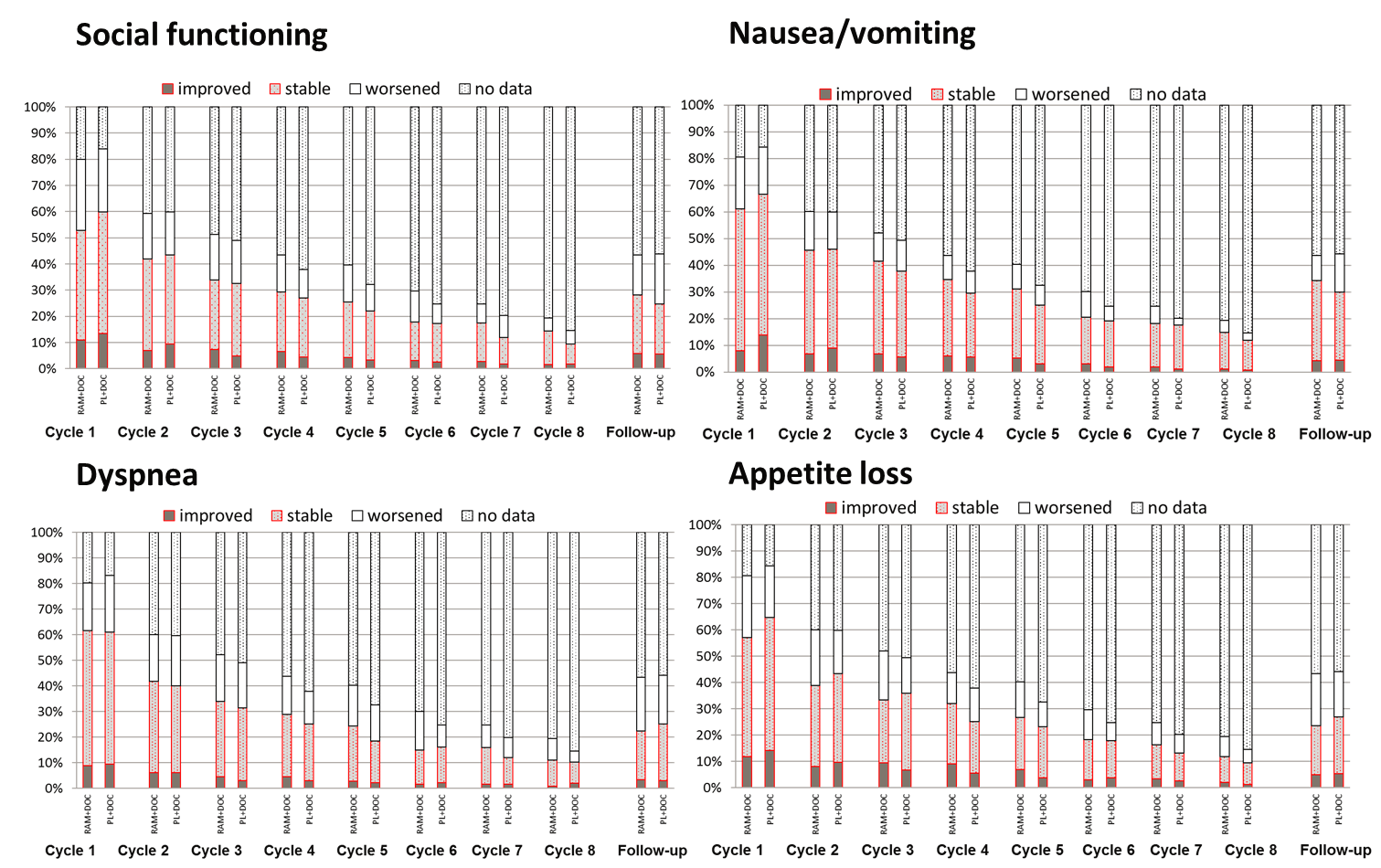
**

**Figure S4 continued**

**
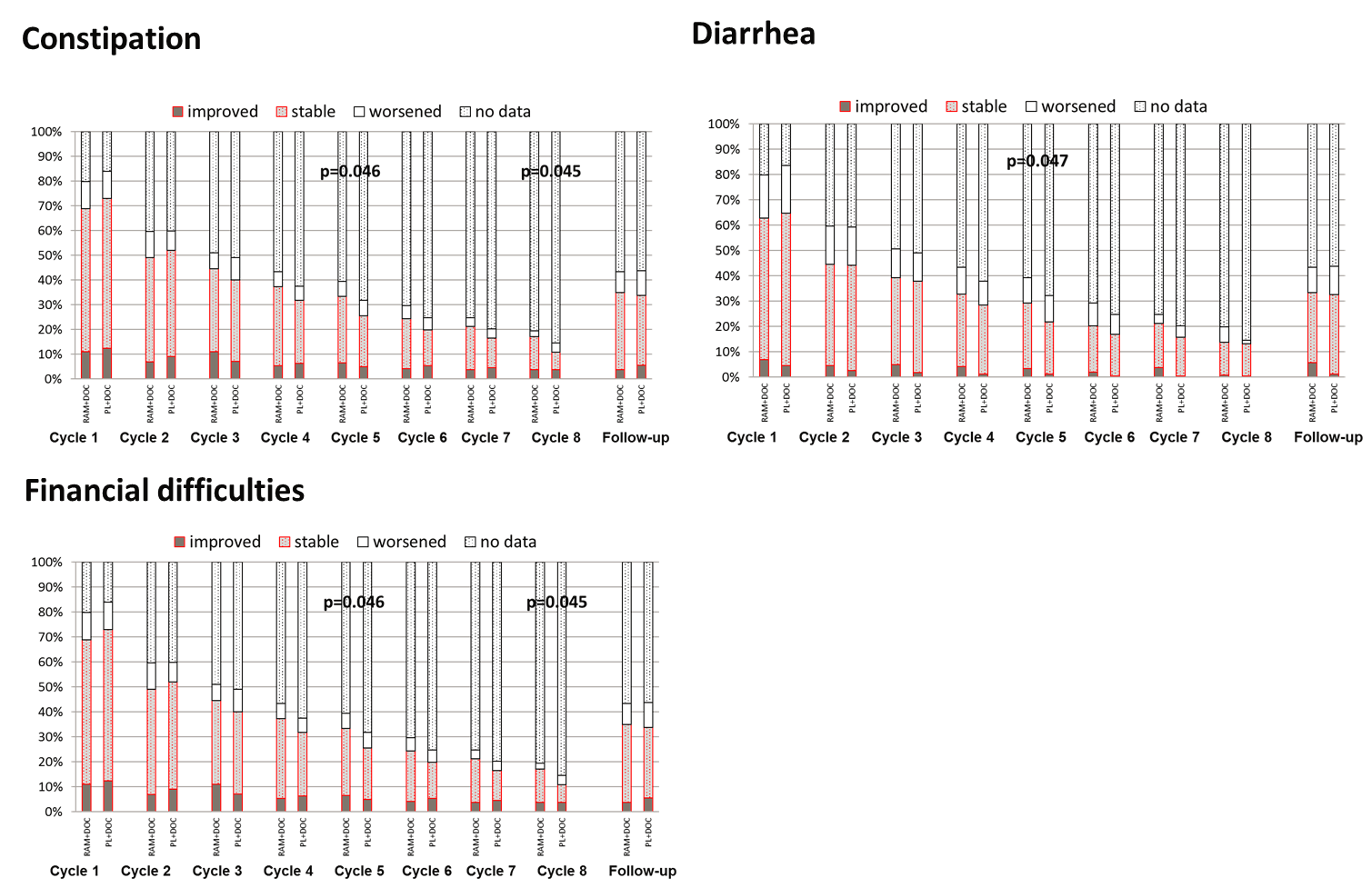
**

DOC = docetaxel; PL = placebo; QLQ-C30 = Cancer Quality of Life Questionnaire C30; QoL = quality of life; RAM = ramucirumab.
